# Supplementary material for: Advanced load frequency control of microgrid using a bat algorithm supported by a balloon effect identifier in the presence of photovoltaic power source
Source: PLoS One. 2023 Oct 20;18(10):e0293246. doi: 10.1371/journal.pone.0293246 (PMC10588904; doi:10.1371/journal.pone.0293246)
Supplement: Attachment — Submitted filename: Reviewer Comments_01.rtf [file pone.0293246.s001.rtf]

In this study, an adaptive LFC method for power systems has been suggested to mitigate the unpredictability of the majority of green energy sources. For the tuning task, an artificial bat algorithm (BA) is utilized and is supported by the balloon effect (BE) identifier. The standard integral controller and Jaya+BE, two more optimization techniques, have been compared with the suggested BA+BE strategy. As per the results and by claimed, the suggested technique BA+BE has a significant advantage over other techniques in terms of maintaining frequency stability in the presence of step/random disturbances and PV source. The study of the paper is needing an improvement. It needs a major change in the revised manuscript. 
(1)	The abstract does not provide a clear problem statement subjected to the current study that the proposed algorithm is trying to solve. The abstract should highlight the significance of the problem and how the proposed algorithm addresses it.
(2)	The abstract lacks details about the proposed algorithm. The abstract should provide some technical details on how the proposed algorithm works and what are the key features that make it different from the existing methods.
(3)	The abstract could benefit from a more concise and straightforward writing style. The abstract should aim to convey the main ideas and findings of the paper in a clear and concise manner.
(4)	In the introduction, the motivation of the paper needs to be explained for more clearly. If possible, give separate sub-section.
(5)	The quality of each figure needs to be improved.
(6)	It is requested to add a mathematical problem formulation. 
(7)	Why only an integral controller is taken into study. If possible, use PID controller for better results.
(8)	What is the range of controller gains has been taken into the optimization task. It should be mentioned and how these parameters are determined, need to explain.
(9)	 Why the authors have chosen BA+BE particularly in this work, the details are missing whether this solution is an optimum (local or global). The simple comparisons shown in the manuscript are believed to be not sufficient.
(10)	 In this work, the test case should be extended to two-area power system and show the LFC response profile.
(11)	 The authors need to go through the entire manuscript to double-check the accuracy/consistency of each equation, table, figure, and reference and to ensure that English grammar errors are avoided.
(12)	 Furthermore, where are the limitations of your study? Clarifying the study's limitations allows the readers to better understand under which conditions the results should be interpreted.
(13)	 In terms of conclusion/implications, did your study suggest a need for further research, what might this consist of, and how might such research extend or improve the current state of knowledge in this field? Are there any practical implications that need to be addressed? All these were not highlighted in the concluding remarks.
(14)	 The discussion of the results needs to include the strengths and weaknesses of the proposed algorithm.
