# Supplementary material for: Advanced load frequency control of microgrid using a bat algorithm supported by a balloon effect identifier in the presence of photovoltaic power source
Source: PLoS One. 2023 Oct 20;18(10):e0293246. doi: 10.1371/journal.pone.0293246 (PMC10588904; doi:10.1371/journal.pone.0293246)
Supplement: Attachment — Submitted filename: Reviewer_Comments.docx [file pone.0293246.s003.docx]

**Reviewers Comments:**

In this work, an adaptive load frequency control (LFC) method for power systems has been studied and suggested to mitigate the frequency deviation problem. Here, bat algorithm (BA) is used to address the LFC issue. For online gain tuning, an integral controller using an artificial BA is utilized, and this control method is supported by a modification known as the balloon effect (BE) identifier. Also, Stability and robustness of analysis of the suggested BA+BE scheme is investigated. In order to validate the MATLAB simulation results, real-time simulation tests are given utilizing a PC and a QUARC pid_e data acquisition card. The presentation and work of this paper is well convincing. Some logical outcome has come out of the paper. The obtained results show the growth and understanding in the field of LFC optimization. I have the acceptance of this paper in the present form.
